# Exosome‐derived miR‐210 involved in resistance to osimertinib and epithelial–mesenchymal transition in *EGFR* mutant non‐small cell lung cancer cells

**DOI:** 10.1111/1759-7714.13943

**Published:** 2021-05-03

**Authors:** Kakeru Hisakane, Masahiro Seike, Teppei Sugano, Akiko Yoshikawa, Kuniko Matsuda, Natsuki Takano, Satoshi Takahashi, Rintaro Noro, Akihiko Gemma

**Affiliations:** ^1^ Department of Pulmonary Medicine and Oncology Graduate School of Medicine, Nippon Medical School Tokyo Japan

**Keywords:** drug resistance, epithelial–mesenchymal transition, exosome, microRNA, osimertinib

## Abstract

**Background:**

Osimertinib is a third‐generation epidermal growth factor receptor‐tyrosine kinase inhibitor (EGFR‐TKI) approved for the treatment of patients with *EGFR*‐mutant non‐small cell lung cancer (NSCLC). However, the mechanisms of acquired drug resistance to osimertinib have not as yet been clarified. Exosomes and microRNAs (miRNAs) are involved in carcinogenesis and drug resistance in human cancers.

**Methods:**

We used previously established osimertinib‐resistant HCC827 (HCC827‐OR) and PC‐9 (PC‐9‐OR) cells. We evaluated the profiles of exosomal miRNA associated with resistance to osimertinib in *EGFR*‐mutant NSCLC cells.

**Results:**

Epithelial–mesenchymal transition (EMT) phenomenon was observed in HCC827‐OR and PC‐9‐OR cells. Microarray and quantitative reverse transcription‐polymerase chain reaction analysis revealed that miR‐210‐3p was co‐upregulated in exosomes isolated from HCC827‐OR and PC‐9‐OR cells compared with those isolated from parental HCC827 and PC‐9 cells. HCC827‐OR cell‐derived exosomes induced EMT changes and resistance to osimertinib in HCC827 cells. Subsequently, the induction of miR‐210‐3p directly promoted the EMT phenomenon and resistance to osimertinib in HCC827 cells.

**Conclusions:**

Exosomal miR‐210‐3p may play a crucial role in resistance to osimertinib in the tumor microenvironment of *EGFR*‐mutant NSCLC.

## Graphical abstract

MiR‐210‐3p was overexpressed in exosomes isolated from osimertinib‐resistant cells. Exosomes derived from osimertinib‐resistant cells induced EMT changes and drug resistance in osimertinib‐sensitive cells. Induction of miR‐210‐3p directly promoted the EMT phenomenon and drug resistance in osimertinib‐sensitive cells.

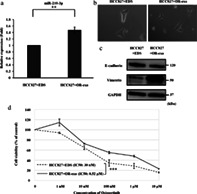

## INTRODUCTION

Osimertinib is a third‐generation epidermal growth factor receptor‐tyrosine kinase inhibitor (EGFR‐TKI) originally designed to target EGFR‐sensitizing mutations and a T790M‐resistant mutation. The latter is the most common mechanism of acquired resistance mechanism to first‐ and second‐generation EGFR‐TKIs, such as gefitinib, erlotinib, and afatinib.^1^ Based on the positive results of the AURA3 study, osimertinib was first approved for the treatment of patients with EGFR T790M mutation‐positive non‐small cell lung cancer (NSCLC) in progression after EGFR‐TKI therapy.^2^ Subsequently, the FLAURA study demonstrated that osimertinib was more efficient than first‐generation EGFR‐TKIs and was rapidly approved as a first‐line treatment of advanced *EGFR*‐mutant NSCLC, regardless of T790M mutation status.^3^ Despite its robust efficacy in first‐ and second‐line settings, patients inevitably develop acquired resistance to osimertinib as well as conventional EGFR‐TKIs. Several molecular mechanisms of acquired resistance to osimertinib have been reported, such as loss of T790M mutation, the acquisition of EGFR mutations (e.g., C797S), activation of a bypass pathway (e.g., MET amplification), HER2 amplification, KRAS mutation, BRAF mutation, PIK3CA mutation and RET fusion, and small cell lung cancer transformation.^4^, ^5^ However, overcoming acquired resistance to osimertinib remains a critical issue to be resolved.

Exosomes are extracellular vesicles with sizes ranging 30–150 nm. They are secreted by various cell types and can promote intercellular communication by transferring varieties of cargos, such as nucleic acid, proteins, and metabolites.^6^, ^7^ MicroRNAs (miRNAs), a family of small non‐coding RNA molecules of approximately 19–25 nucleotides in length, are essential cargos delivered by exosomes.^7^ We have previously identified miRNAs associated with *EGFR*‐mutant NSCLC, such as miR‐21 and miR‐134/487b/655 clusters.^8^, ^9^ Epithelial–mesenchymal transition (EMT) induces tumor progression and drug resistance to various cytotoxic and targeted drugs, including EGFR‐TKIs.^10^ Recently, the role of exosomal miRNAs as an inducer of EMT and acquired drug resistance in cancer has been gradually elucidated.^7^, ^11^, ^12^, ^13^ Several studies have reported that exosomal miRNAs are associated with acquired resistance to first‐generation EGFR‐TKIs.^14^, ^15^, ^16^ However, the involvement of exosomal miRNAs in the development of EMT and resistance to third‐generation EGFR‐TKIs remains unclear.

In this study we evaluated the expression profile of exosomal miRNAs in osimertinib‐sensitive and ‐resistant cells to identify the potential associations of exosomes with the development of resistance to osimertinib in *EGFR*‐mutant NSCLC.

## METHODS

### Cell lines and cell culture

Four *EGFR*‐mutant lung adenocarcinoma cell lines were used in this study. HCC827 cells (with a deletion in exon 19) were purchased from the American Type Culture Collection and PC‐9 cells (with a deletion in exon 19) were obtained from Immuno‐Biological Laboratories. These cells were obtained from 2010 to 2016. Osimertinib‐resistant HCC827 (HCC827‐OR) and osimertinib‐resistant PC‐9 (PC‐9‐OR) cell lines were established using a stepwise method, as previously described.^17^ Cells were amplified and frozen, and one aliquot of each was thawed for this research. All cells were routinely screened for the absence of mycoplasma and cultured in RMPI 1640 medium (FUJIFILM Wako Pure Chemical Co.) with 10% heat‐inactivated fetal bovine serum (FBS) (BioWest) and 1% penicillin and streptomycin (FUJIFILM Wako Pure Chemical Co) at 37°C in a 5% CO_2_ incubator. Falcon Cell Culture Inserts (Corning Inc.) with a transparent polyethylene terephthalate membrane (pore size: 0.4 μm) were used for co‐culture. HCC827 and PC‐9 cells were plated into six‐well plates (2 × 10^5^ cells/well), the device was placed on the six‐well plates, and equal numbers of their respective resistant cells were plated into the device. After 72 h, the device was removed and HCC827 and PC‐9 cells were used for protein extraction.

### Exosome isolation and identification

At approximately 100% confluency, we washed the cells with phosphate‐buffered saline (PBS) and incubated in medium without FBS. After 48 h, the culture supernatant was harvested, and clarified by vacuum filtration using a Steriflip filter unit (#SCGP00525; 0.22 μm Millipore Express PLUS [PES] membrane). For the device equilibration, 2 ml of PBS was added to Equilibrate Amicon Ultra‐15 filter (#UFC901024, 10 kDa molecular weight cutoff; Merck Millipore), centrifuged at 4000 × *g* for 10 min, and removed from the filter device. Approximately 13–15 ml of the clarified sample was added to the filter device and centrifuged at 4000 × *g* for 30 min for concentration. Any unconcentrated sample at the bottom of the device was aspirated. Next, approximately 3–5 ml of PBS was added to the concentrated sample in the collection tube, and the solution was centrifuged at 4000 × *g* for 30 min for buffer exchange. Subsequently, the concentrated sample was recovered from the filter device. Protein was isolated from the extracted exosome samples and used for western blotting analysis to detect exosome‐specific markers CD63 and TSG101. The aliquots were stored at 4°C and sent to FUJIFILM Wako Pure Chemical Co. for observation via transmission electron microscopy (TEM) and nanoparticle tracking analysis (NTA) for the identification of exosomes. Exosome samples were also used for RNA extraction and cell treatment.

### Drugs and growth‐inhibition assay

Osimertinib was purchased from Selleck Chemicals. Growth inhibition was assessed by the MTS assay to examine the effect of osimertinib on the four NSCLC cell lines. Cell suspensions (5 × 10^3^ cells/well) were seeded into 96‐well plates and various concentrations of osimertinib or vehicle (dimethyl sulfoxide) were added. After incubation at 37°C for 72 h, MTS was added to each well and incubated at 37°C for 1.5 h, after which absorbance was measured using a microplate reader with a test wavelength of 450 nm. The IC_50_ value was defined as the concentration required for 50% reduction of the growth.

### Western blotting analysis

Protein extraction, two dimensional‐polyacrylamide gel electrophoresis, and transfer to nitrocellulose membranes were performed as previously described.^17^, ^18^ Antibodies against E‐cadherin (1:5000), β‐actin (1:5000), GAPDH (1:5000), CD63 (1:5000), and TSG101 (1:5000) were purchased from Santa Cruz Biotechnology. Antibodies against vimentin (1:2000), EGFR (1:5000), phosphorylated‐EGFR (p‐EGFR) (1:5000), AKT (1:5000), p‐AKT (1:5000), ERK (1:2000), and p‐ERK (1:5000) were obtained from Cell Signaling Technology.

### RNA extraction and miRNA microarray analysis

Total RNA was extracted using TRIzol reagent (Thermo Fisher Scientific) from cells and isolated exosomes, as previously described.^19^ MiRNA analysis was performed using 3D‐Gene Human miRNA oligo chips version 21 (TORAY). Microarray data have been deposited in NCBI's Gene Expression Omnibus (GEO; http://www.ncbi.nlm.nih.gov/geo/) and are accessible through the GEO series accession number GSE 165540.

### Real‐time quantitative reverse transcription‐polymerase chain reaction (qRT‐PCR)

For qRT‐PCR, the expression levels of miRNAs were measured using a Taqman MicroRNA Assay (Thermo Fisher Scientific) and a 7500 fast real‐time PCR system, as previously described.^8^ The miRNA levels of cells were normalized against RNU66, while the miRNA levels of exosomes were normalized against U6 small nuclear RNA and HY3. MiRNA expression was quantified using the 2^‐ΔΔCt^ method.^20^

### Oligonucleotide transfection

After 24 h of cell seeding, cells were transfected for 24 h in Opti‐MEM Reduced Serum Medium (Thermo Fisher Scientific) using a miR‐210‐3p mimic (Thermo Fisher Scientific), negative control (Thermo Fisher Scientific), and Lipofectamine 2000 (Thermo Fisher Scientific) according to the instructions provided by the manufacturer. The mimic complexes were transfected into cells at a final concentration of 50 nM. The transfection medium was replaced with fresh medium 24 h later, and cells were incubated for another 48 h. Transforming growth factor‐β1 (TGF‐β1) was obtained from R&D Systems, Inc. Cells were exposed to 5 ng/ml TGF‐β1 for 48 h.

### Statistical analysis

The standard Student *t*‐test was used to determine the significance of differences compared with the control group. *p* < 0.05 denoted statistically significant difference. All the statistical analyses were performed using the JMP statistical software package for Windows, version 11 (SAS Institute).

## RESULTS

### Characteristics of HCC827‐OR and PC‐9‐OR cells

**FIGURE 1:**
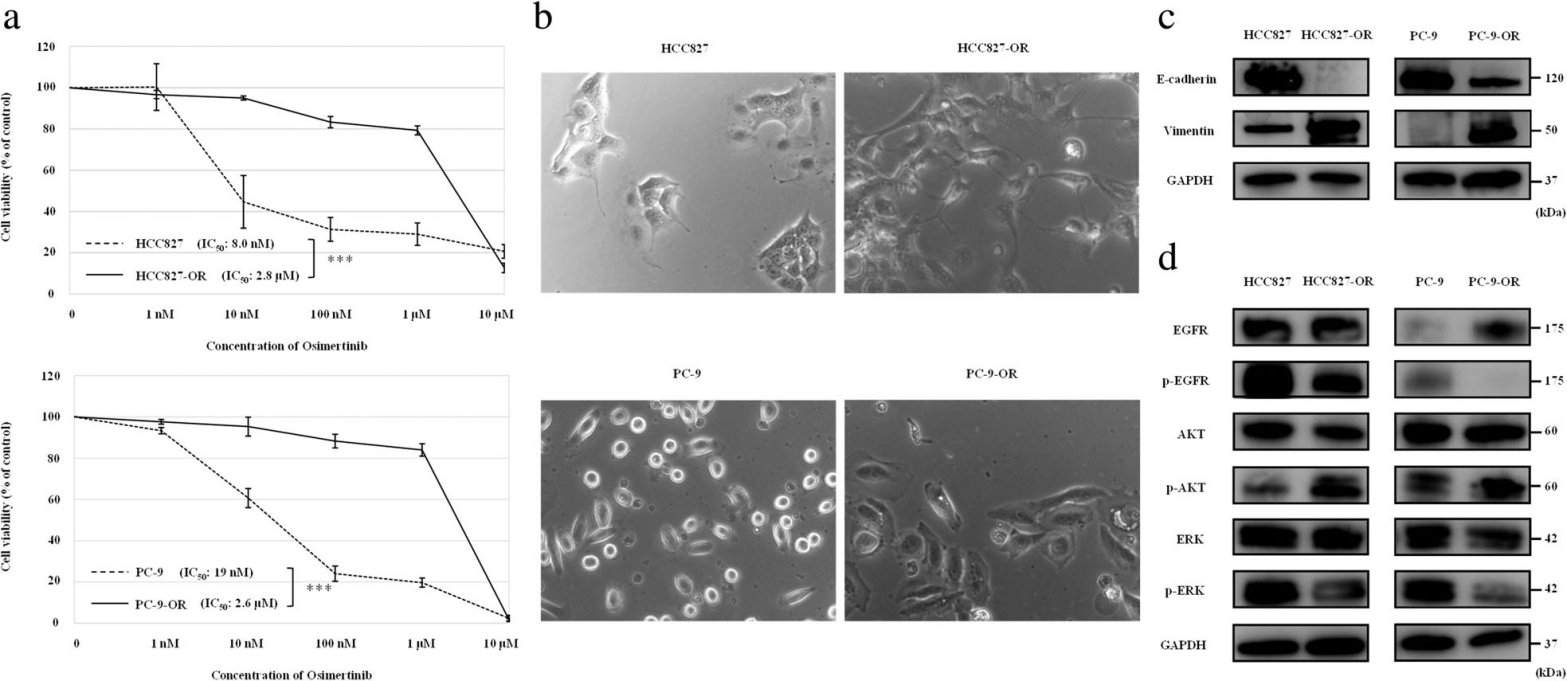
Osimertinib‐resistant HCC827 and PC9 cells. (a) Results of cell viability assays. Data are expressed as the mean ± SE from three independent experiments. ***p < 0.001. (b) Four *EGFR*‐mutant NSCLC cells observed under a light microscope. Osimertinib‐resistant HCC827 (HCC827‐OR); Osimertinib‐resistant PC‐9 (PC‐9‐OR). (c) Protein expression of factors related to EMT, as shown by western blotting analysis. (d) Protein expression of EGFR signal pathway molecules. EGFR, epidermal growth factor receptor; EMT, epithelial–mesenchymal transition; ERK, extracellular signal‐regulated kinase; GAPDH, glyceraldehyde‐3‐phosphate dehydrogenase; IC_50_, concentration needed for 50% reduction of the growth; NSCLC, non‐small cell lung cancer; p‐EGFR, phosphorylated‐EGFR; SE, standard error

We first evaluated the features of four *EGFR*‐mutant NSCLC cell lines. The IC_50_ values of osimertinib for these cells are shown in Figure 1a. Both HCC827‐OR and PC‐9‐OR cells were resistant to osimertinib. Morphological changes from epithelial to spindle‐type mesenchymal cells were observed in HCC827‐OR and PC‐9‐OR cells compared with the respective parental cells (Figure 1b). Lower and higher expression levels of E‐cadherin and vimentin, respectively, were shown in HCC827‐OR and PC‐9‐OR cells (Figure 1c). These findings suggested the presence of the EMT phenomenon in osimertinib‐resistant cells. We performed western blotting analysis to determine whether the osimertinib‐resistant cells sustained the activation of the EGFR signaling pathway (Figure 1d). Decreased and increased expression of EGFR and AKT phosphorylation, respectively, were observed in both HCC827‐OR and PC‐9‐OR cells. These findings suggest the involvement of the EGFR signal‐independent pathway in the mechanism of resistance to osimertinib in these cells.

### Co‐culture with osimertinib‐resistant cells induces EMT changes in osimertinib‐sensitive cells

**FIGURE 2:**
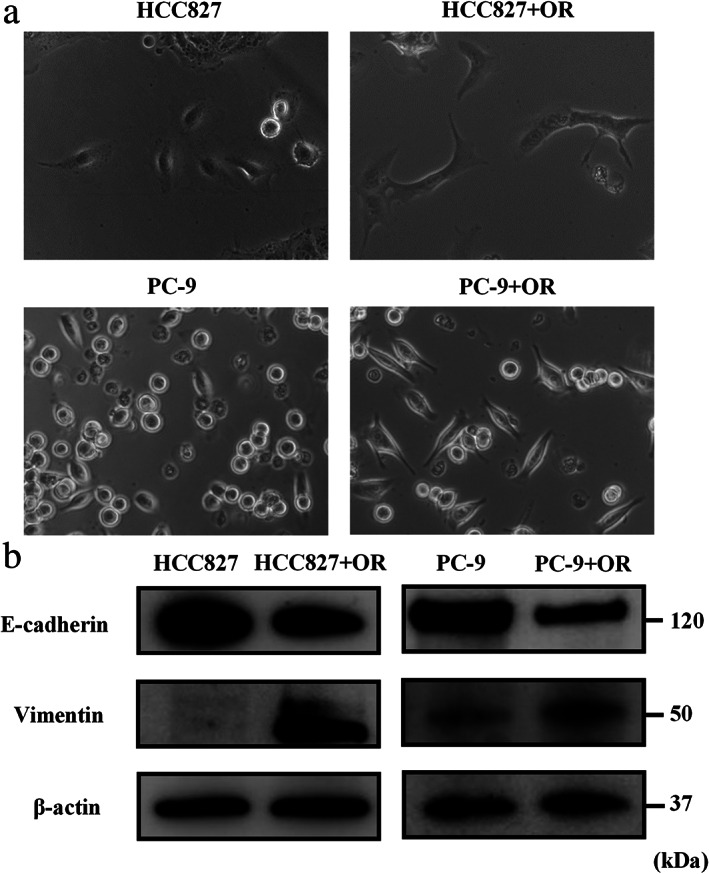
HCC827 and PC‐9 cells co‐cultured with their respective osimertinib‐resistant cells. (a) HCC827 cells, HCC827 cells cocultured with HCC827‐OR cells (HCC827 + OR), PC‐9 cells, and PC‐9 cells co‐cultured with PC‐9‐OR cells (PC‐9 + OR) observed under a light microscope. (b) Protein expression of factors related to EMT, as shown by western blotting analysis. EMT, epithelial–mesenchymal transition; HCC827‐OR, osimertinib‐resistant HCC827; PC‐9‐OR, osimertinib‐resistant‐PC‐9

HCC827 and PC‐9 cells were cocultured with their respective osimertinib‐resistant cells to determine whether the osimertinib‐resistant cells exert a potential effect on the parental cells. Morphological changes from epithelial to spindle‐type mesenchymal cells were observed in HCC827 and PC‐9 cells cocultured with HCC827‐OR (HCC827 + OR) and PC‐9‐OR cells (PC‐9 + OR), respectively (Figure 2a). Western blotting analysis revealed increased and decreased expression of vimentin and E‐cadherin, respectively, in HCC827 + OR and PC‐9 + OR (Figure 2b). These findings demonstrated that coculture with osimertinib‐resistant cells results in the transfer of factors associated with the EMT of osimertinib‐resistant cells to the parental cells.

### MiR210‐3p is upregulated in exosomes derived from osimertinib‐resistant cells

**FIGURE 3:**
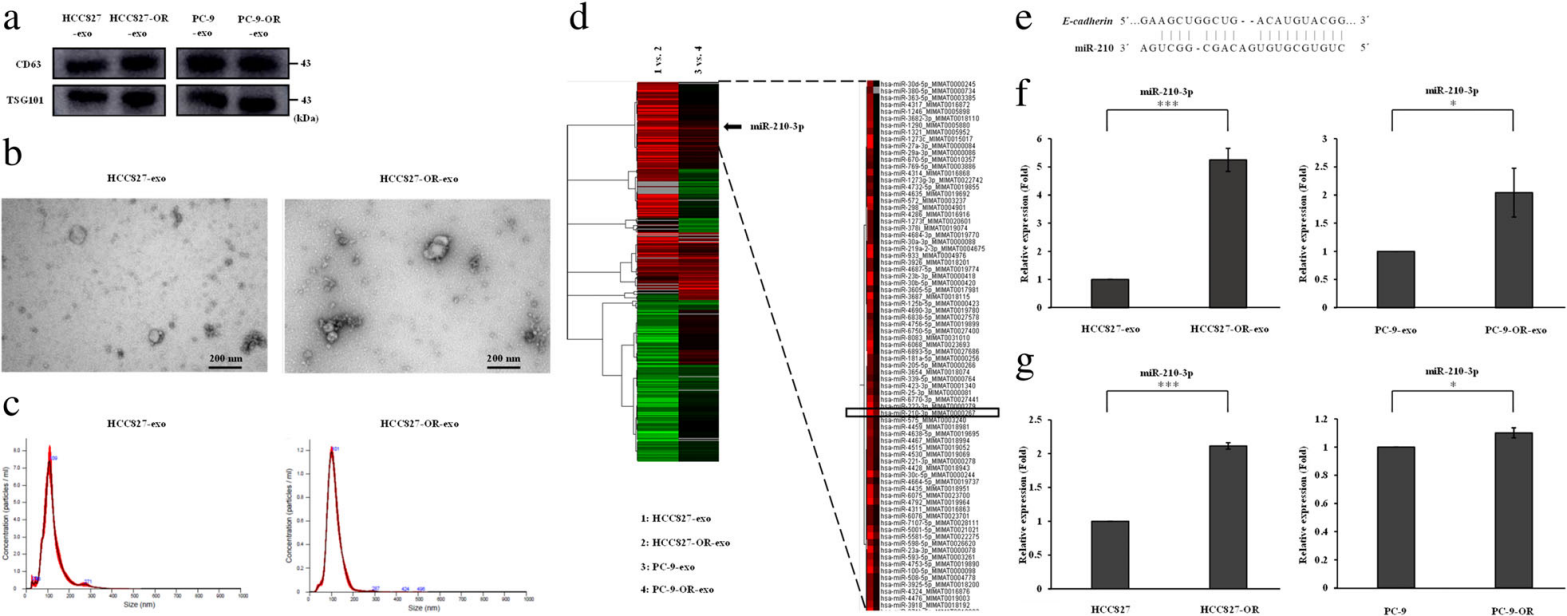
Characterization of exosomes derived from NSCLC cell lines and miRNA microarray analysis. (a) Protein expression of exosome‐specific markers, as shown by western blotting analysis. Exosomes derived from HCC827 cells (HCC827‐exo); exosomes derived from HCC827‐OR cells (HCC827‐OR‐exo); exosomes derived from PC‐9 cells (PC‐9‐exo); exosomes derived from PC‐9‐OR cells (PC‐9‐OR‐exo). (b) Representative TEM images of exosomes. (c) NTA analysis of the size distribution of exosomes. (d) Heatmap displays the differentially expressed miRNAs between osimertinib‐resistant exosomes and osimertinib‐sensitive exosomes. (e) MiR‐210 binding sites in E‐cadherin, as determined by bioinformatic analysis.^22^ (f) Exosomal miR‐210‐3p expression, as measured by qRT‐PCR analysis. Data are expressed as the mean ± SD from three independent experiments. *p < 0.05, ***p < 0.001. (g) Cellular miR‐210‐3p expression, as detected by qRT‐PCR analysis. Data are expressed as the mean ± SD from three independent experiments. *p < 0.05, ***p < 0.001. HCC827‐OR, osimertinib‐resistant HCC827; miRNA, microRNA; NSCLC, non‐small cell lung cancer; NTA, nanoparticle tracking analysis; PC‐9‐OR, osimertinib‐resistant‐PC‐9; qRT‐PCR, quantitative reverse transcription‐polymerase chain reaction; SD, standard deviation; TEM, transmission electron microscope

Exosomes were isolated from the culture supernatants of four NSCLC cell lines by a centrifugal ultrafiltration‐based method to investigate the role of exosomes in EMT and resistance to osimertinib in *EGFR*‐mutant NSCLC cells. We confirmed the enrichment of exosomal proteins, such as CD63 and TSG101, by western blotting analysis (Figure 3a). The representative micrograph obtained by TEM revealed vesicles with a round or oval membrane (Figure 3b). NTA indicated that the diameter of the vesicles ranged from 40 to 200 nm and the peak diameters of the vesicles were approximately 100 nm (Figure 3c). These results suggested that the exosomes were successfully isolated. Subsequently, miRNA microarray analysis was performed to identify the candidate exosomal miRNAs causing EMT and resistance to osimertinib in NSCLC cells. Figure 3d shows that the expression profiles of miRNAs differed between exosomes derived from HCC827 cells (HCC827‐exo) and HCC827‐OR cells (HCC827‐OR‐exo) and those derived from PC‐9 cells (PC‐9‐exo) and PC‐9‐OR cells (PC‐9‐OR‐exo), based on the criteria of | Log2 ratio | ≥ 2. After observing the co‐upregulation of miRNAs in HCC827‐OR‐exo and PC‐9‐OR‐exo, we focused on miR‐210‐3p, which correlates with EMT in several malignancies.^21^, ^22^, ^23^ There were no miRNAs associated with EMT, except for miR‐210‐3p. Using RNAhybrid tools, it was shown that miR‐210 could directly target the E‐cadherin open reading frames region (Figure 3e).^22^ We confirmed that the relative expression levels of miR‐210‐3p were significantly higher in HCC827‐OR‐exo and PC‐9‐OR‐exo than in HCC827‐exo and PC‐9‐exo, respectively (Figure 3f). In addition, miR‐210‐3p expression was significantly increased in HCC827‐OR and PC‐9‐OR cells compared with HCC827 and PC‐9 cells, respectively (Figure 3g). From these results, it was concluded that exosomal miR‐210‐3p may be involved in EMT and resistance to osimertinib in *EGFR*‐mutant NSCLC cells. The differences in miR‐210‐3p expression between PC‐9‐exo and PC‐9‐OR‐exo, as well as between PC‐9 and PC‐9‐OR cells, were relatively small. Hence, HCC827 and HCC827‐OR cells were used for the subsequent experiments.

### HCC827‐OR cell‐derived exosomes induce EMT changes and resistance to osimertinib in HCC827 cells

**FIGURE 4:**
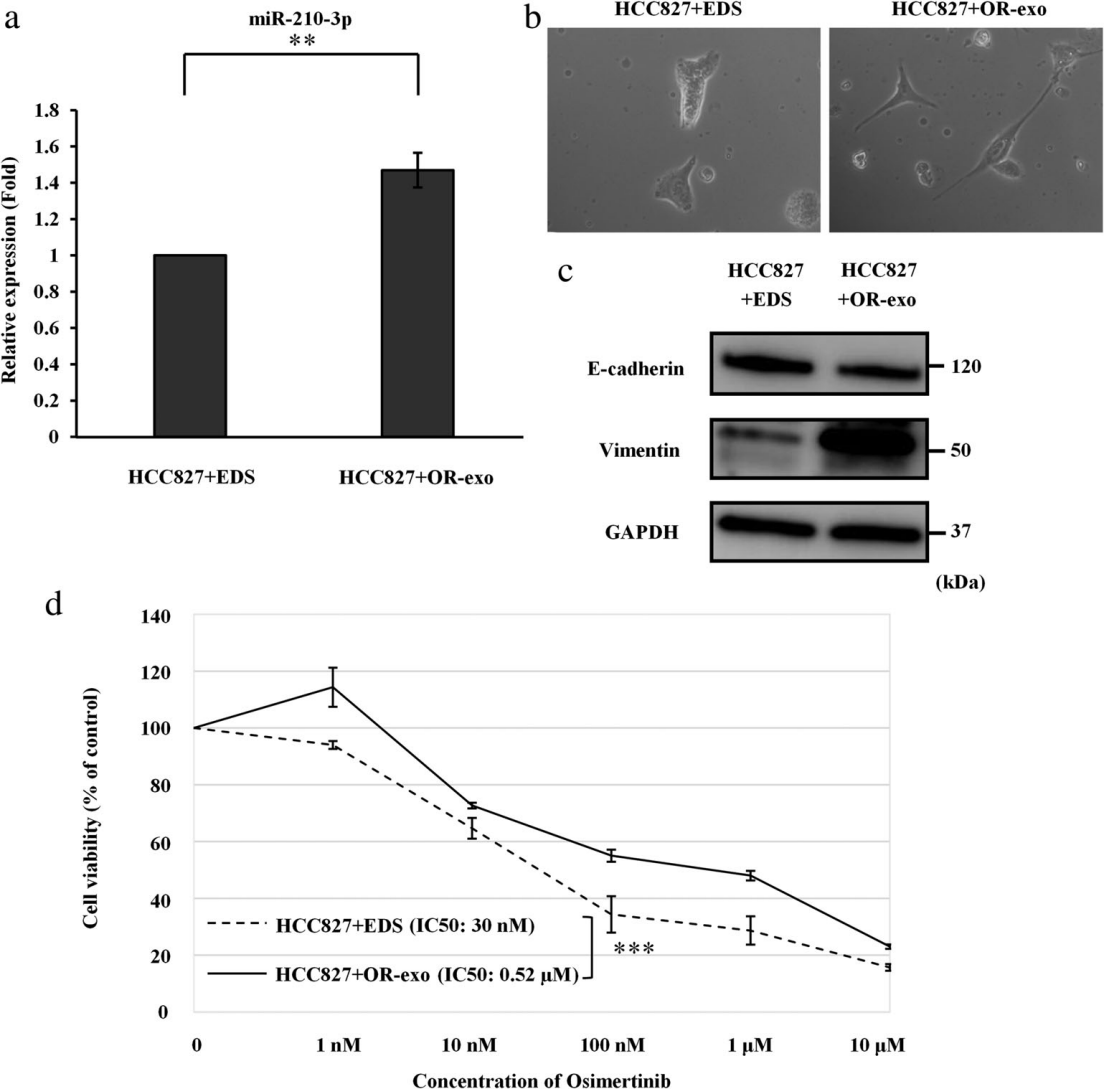
The effect of exosomes derived from HCC827‐OR on HCC827 cells. (a) MiR‐210‐3p expression in HCC827 cells after treatment with exosome‐depleted supernatant (EDS) (HCC827 + EDS) and HCC827‐OR‐exo (HCC827 + OR‐exo), as determined by qRT‐PCR analysis. Data are expressed as the mean ± SD from three independent experiments. **p < 0.01. (b) HCC827 + EDS and HCC827 + OR‐exo observed under a light microscope. (c) Protein levels of EMT markers, as shown by western blotting analysis. (d) The results of the cell viability assays are shown. Data are expressed as the mean ± SE from three independent experiments. *** *p* < 0.001. EMT, epithelial–mesenchymal transition; GAPDH, glyceraldehyde‐3‐phosphate dehydrogenase; HCC827‐OR, osimertinib‐resistant HCC827; HCC827‐OR‐exo, exosomes derived from HCC827‐OR cells; IC_50_, concentration needed for 50% reduction of the growth; qRT‐PCR, quantitative reverse transcription‐polymerase chain reaction; SD, standard deviation; SE, standard error

HCC827‐OR‐exo (100 μg/ml) was administered to HCC827 cells to elucidate the effect of exosomes derived from osimertinib‐resistant cells on osimertinib‐sensitive cells. Exosome‐depleted supernatant (EDS) was used as the control. Figure 4a shows that miR‐210‐3p was upregulated in HCC827 cells treated with HCC827‐OR‐exo compared with HCC827 cells treated with EDS. These results suggested that exosomal miR‐210‐3p derived from HCC827‐OR cells was transferred into HCC827 cells. HCC827‐OR‐exo induced the EMT phenomenon in HCC827 cells (Figure 4b). Increased and decreased levels of vimentin and E‐cadherin, respectively, were observed in HCC827 cells incubated with HCC827‐OR‐exo, as shown by western blotting analysis (Figure 4c). The IC_50_ value of osimertinib was significantly higher for HCC827 cells treated with HCC827‐OR‐exo than that for the control (Figure 4d). These data indicated that HCC827‐OR cell‐derived exosomes could promote EMT and resistance to osimertinib in HCC827 cells, and exosomal miR‐210‐3p may play an important role in these mechanisms.

### MiR‐210‐3p affects the EMT phenomenon and resistance to osimertinib in HCC827 cells

**FIGURE 5:**
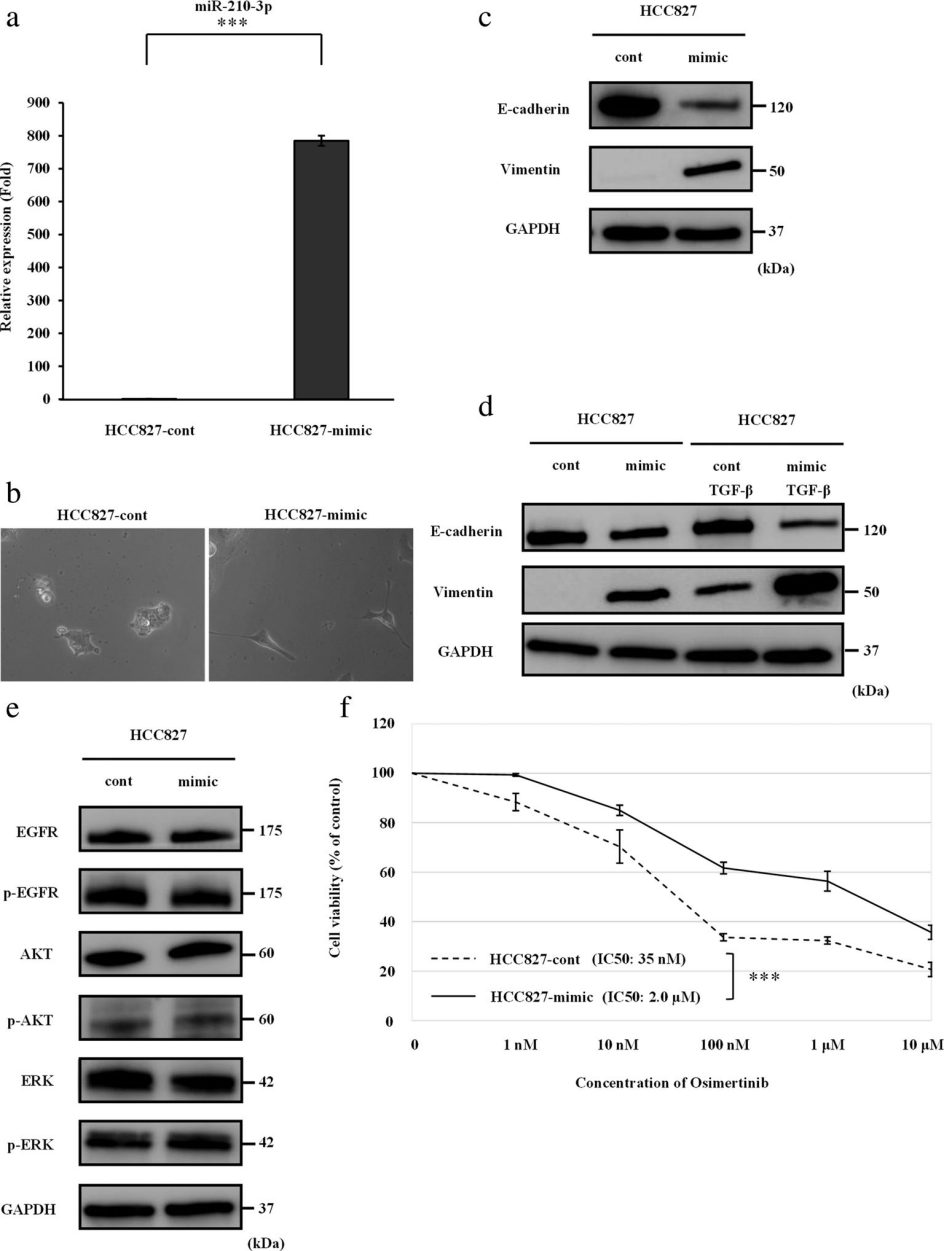
MiR‐210‐3p promotes EMT and resistance to osimertinib in HCC827 cells. (a) MiR‐210‐3p expression in HCC827 cells after transfection of the miR‐210‐3p mimic, as determined by qRT‐PCR analysis. Control mimic (cont); miR‐210‐3p mimic (mimic). Data are expressed as the mean ± SD from three independent experiments. ***p < 0.001. (b) HCC827‐cont cells and HCC827‐mimic cells observed under a light microscope. (c) Protein levels of EMT markers in HCC827 cells after transfection of miR‐210‐3p mimic, as shown by western blotting analysis. (d) Protein levels of EMT markers in HCC827 cells after transfection of the miR‐210‐3p mimic with or without TGF‐β stimulation. (e) Protein expression of EGFR signaling pathway molecules in HCC827 cells after transfection of the miR‐210‐3p mimic. (f) The results of the cell viability assays are shown. Data are expressed as the mean ± SE from three independent experiments. ***p < 0.001. EGFR, epidermal growth factor receptor; EMT, epithelial–mesenchymal transition; ERK, extracellular signal‐regulated kinase; GAPDH, glyceraldehyde‐3‐phosphate dehydrogenase; IC_50_, concentration needed for 50% reduction of the growth; p‐EGFR, phosphorylated‐EGFR; qRT‐PCR, quantitative reverse transcription‐polymerase chain reaction; SD, standard deviation; SE, standard error; TGF‐β, transforming growth factor‐β

Next, a miR‐210‐3p mimic was transfected into HCC827 cells to clarify the involvement of miR‐210‐3p in EMT and resistance to osimertinib in *EGFR*‐mutant NSCLC cells. After transfection of the miR‐210‐3p mimic in HCC827 cells, the relative expression levels of miR‐210‐3p were significantly higher than those detected in HCC827 cells transfected with miR‐210‐3p control (Figure 5a). The miR‐210‐3p mimic induced the EMT phenomenon in HCC827 cells (Figure 5b). Western blotting analysis showed increased and decreased levels of vimentin and E‐cadherin, respectively, in HCC827 cells after transfection of the miR‐210‐3p mimic (Figure 5c). Moreover, miR‐210‐3p suppressed the expression of E‐cadherin and promoted that of vimentin with or without TGF‐β stimulation (Figure 5d). The analysis showed that miR‐210‐3p was not involved in the EGFR signaling pathway (Figure 5e). The IC_50_ value of osimertinib was significantly higher for HCC827 cells transfected with the miR‐210‐3p mimic than for HCC827 cells transfected with miR‐210‐3p control (Figure 5f). These results suggested that miR‐210‐3p could induce EMT and resistance to osimertinib in HCC827 cells independently of the EGFR signaling pathway.

## DISCUSSION

In this study, we found that osimertinib‐resistant, *EGFR*‐mutant NSCLC cells could produce exosomes with abundant miR‐210‐3p promoting EMT and drug resistance in osimertinib‐sensitive cells.

Exosomes could be shed from drug‐resistant cells, transferring mediators of drug resistance to drug‐sensitive cells and leading to the acquisition of the cancer drug‐resistant phenotype.^24^ These mediators include miRNAs, long non‐coding RNAs (lncRNAs), and proteins such as drug‐efflux pumps. Furthermore, several miRNAs and proteins delivered from exosomes have been reported as EMT regulators in a variety of cancers.^13^ In a human bronchial epithelial cell model, mesenchymal NSCLC‐derived exosomes from chemoresistant cells were capable of inducing EMT and drug resistance to chemosensitive epithelial cells.^25^ Moreover, exosomes derived from chemoresistant NSCLC cells increased the chemoresistance of chemosensitive cells via delivery of miRNAs, such as miR‐100‐5p and miR‐223‐3p.^26^, ^27^ In *EGFR*‐mutant NSCLC, exosomal miRNAs (e.g., miR‐21, miR‐214, and miR‐522‐3p) contribute to the development of resistance to gefitinib.^14^, ^15^, ^16^ Recently, it was reported that exosomal lncRNA is associated with resistance to osimertinib in *EGFR*‐mutant NSCLC.^28^ The present study showed that osimertinib‐resistant, *EGFR*‐mutant NSCLC cells with EMT features release exosomes encapsulating resistant components with increased levels of miR‐210, thereby inducing EMT changes and acquired drug resistance in osimertinib‐sensitive cells. This finding is consistent with previous results. Exosomes derived from drug‐resistant cells may play a crucial role in EMT and resistance to osimertinib in the tumor microenvironment of *EGFR*‐mutant NSCLC. Thus far, three novel therapeutic strategies against cancer‐derived exosomes have been proposed: inhibition of cancer cell exosome production; elimination of circulating cancer cell‐derived exosomes; and disruption of cancer cell‐derived exosome uptake by recipient cells.^29^ Currently, there is limited knowledge regarding the molecules involved in these processes, and drugs targeting cancer cell‐derived exosomes have not been approved for the clinical setting. Nevertheless, these strategies are reasonable to overcome the drug resistance mediated by exosomes. Further exploratory studies are warranted to develop a therapeutic strategy for inhibiting exosomes derived from osimertinib‐resistant cells.

We identified that miR‐210‐3p may be a significant cargo delivered by exosomes derived from osimertinib‐resistant NSCLC cells affecting EMT and drug resistance in osimertinib‐sensitive cells. MiR‐210 is involved in numerous biological processes, such as mitochondrial metabolism, angiogenesis, the DNA damage response, cell proliferation, and apoptosis.^30^ MiR‐210 is an oncogenic miRNA. Overexpression of miR‐210 was observed in multiple malignant tumors, including lung cancer.^30^, ^31^, ^32^ Recently, it was reported that miR‐210 promotes EMT in prostate, breast, and pancreatic cancers.^21^, ^22^, ^23^ Exosomes secreted from colon cancer cells containing miR‐210 modify the adhesion dynamic of neighboring metastatic cells and are involved in the EMT process.^33^ Moreover, miR‐210 in exosome secreted from hypoxic bone marrow‐derived mesenchymal stem cells and cancer‐associated fibroblasts could affect the EMT in NSCLC cells.^34^, ^35^ Regarding drug resistance, miR‐210 has been associated with resistance to 5‐fluorouracil in colorectal cancer and gemcitabine in cholangiocarcinoma.^36^, ^37^ Exosomes derived from gemcitabine‐resistant pancreatic cancer stem cells mediate the horizontal transfer of drug‐resistant traits to gemcitabine‐sensitive pancreatic cancer cells by delivering miR‐210.^38^ In this study, miR‐210‐3p was upregulated in exosomes isolated from osimertinib‐resistant NSCLC cells. Moreover, the exosomes rich in miR‐210‐3p promoted EMT and drug resistance in osimertinib‐sensitive NSCLC cells. In addition, miR‐210‐3p directly induced EMT and drug resistance in osimertinib‐sensitive cells. Taken together, exosomal miR‐210‐3p may be a key molecule of EMT and resistance to osimertinib in *EGFR*‐mutant NSCLC cells. The development of nucleic acid drugs has recently been progressing. However, therapeutic agents targeting miRNAs have not as yet been established. The development of therapeutic agents that directly target miR‐210‐3p to inactivate oncogenic properties would have a significant clinical impact.

We also observed that the induction of miR‐210‐3p promoted EMT in HCC827 cells independently of TGF‐β stimulation. TGF‐β is a strong inducer of EMT.^39^, ^40^, ^41^ TGF‐β‐induced EMT has been associated with acquired resistance to EGFR‐TKIs in *EGFR*‐mutant NSCLC.^9^, ^42^, ^43^, ^44^, ^45^ MiR‐210‐3p‐induced EMT may be associated with the development of resistance to osimertinib in *EGFR*‐mutant NSCLC with or without TGF‐β stimulation. Next, the induction of miR‐210‐3p accelerated the development of resistance to osimertinib without affecting the EGFR signaling pathway in HCC827 cells. The miR‐210‐3p‐induced EMT may contribute to resistance to osimertinib as a mechanism involving the bypass pathway. Thus far, several target genes of miR‐210 involved in various biological processes and cellular functions have been identified.^29^ E‐cadherin associated with EMT may be a candidate molecule in our study. E‐cadherin‐targeted miR‐210 promotes breast cancer carcinogenesis under the hypoxic condition.^22^ MiR‐210‐3p promotes EMT and bone metastasis thorough NF‐κB signaling by targeting TNPI1 and SOCS1 in prostate cancer.^21^ MiR‐210 inhibits the expression of HOXA9 to activate the NF‐κB signaling, induces EMT, and reduces the sensitivity of pancreatic cancer cells to gemcitabine induced by HIF1A under hypoxia.^23^ MiR‐210 in exosomes secreted by cancer‐associated fibroblasts promotes EMT by targeting UPF1 through PTEN/PI3K/AKT pathway in NSCLC cells.^35^ The pathway of miR‐210‐induced EMT is complex and may vary depending on the tumor microenvironment.

In summary, we conclude that exosomes derived from osimertinib‐resistant, *EGFR*‐mutant NSCLC cells induced EMT and drug resistance in osimertinib‐sensitive cells via delivery of miR‐210. Exosomal miR‐210 may play an important role in the development of resistance to osimertinib in the tumor microenvironment and serve as a therapeutic target to conquer this resistance in *EGFR*‐mutant NSCLC. We will perform further studies to clarify the effect of exosomal miR‐210 on resistance to osimertinib in *EGFR*‐mutant NSCLC.

## CONFLICT OF INTEREST

Dr A. Gemma received a commercial research grant from AstraZeneka Co. and Boehringer Ingelheim Pharmaceuticals, Inc. and has received speakers'bureau honoraria from AstraZeneka Co. and Boehringer Ingelheim Pharmaceuticals, Inc. Dr M. Seike received a commercial research grant from Chugai Pharmaceutical Co., and Boehringer Ingelheim Pharmaceuticals, Inc. and has received speakers' bureau honoraria from AstraZeneka Co., Ltd., Chugai Pharmaceutical Co., and Boehringer Ingelheim Pharmaceuticals, Inc. The other authors have no potential conflicts of interest.
